# Detection of PCV3 in German wild boars

**DOI:** 10.1186/s12985-019-1133-9

**Published:** 2019-02-22

**Authors:** Carolin Prinz, Milena Stillfried, Lena Katharina Neubert, Joachim Denner

**Affiliations:** 1 Robert Koch Institute, 13353 Berlin, Germany; 2 Department of Ecological Dynamics, Leibniz Institute for Zoo and Wildlife Research, Berlin, Germany

**Keywords:** Porcine circoviruses, PCV3, Wild boars

## Abstract

**Background:**

Porcine circovirus 3 is a newly described circovirus circulating worldwide. PCV3 may play an etiologic role in different pig diseases. Two different genotypes of PCV3 were described, PCV3a and PCV3b. In order to analyse whether PCV3 is also present in wild boars, animals living in and near Berlin were studied. The animals had been analysed previously and were found to form two genetically distinct and geographically coherent clusters.

**Methods:**

To detect PCV3 in wild boars, a PCR was performed, to analyse the virus in detail, parts of the sequence of the capsid protein were sequenced. In addition, a screening for PCV1 and PCV2 was performed using PCR.

**Results:**

For the first time, PCV3 was detected in German wild boars, with 50% of the animals infected in one genetic cluster, and 23% in the second cluster. In both populations which were divided in the years of division of Berlin, PCV3b was detected, in one case also PCV3a was detected. In some animals, co-infections with PCV1 and PCV2 or triple infections were detected.

**Conclusion:**

The data show a high prevalence of PCV3 and co-infections with PCV1 and PCV2 in German wild boars. The finding of PCV3 in both clusters suggests that the virus was introduced into the animal populations before Berlin was divided. Furthermore, the methods used will be indispensable for screening for circoviruses in pigs genetically modified for xenotransplantation.

## Background

Circoviruses are the smallest known replicating viruses, they are characterised by a circular single-stranded DNA genome with less than 2000 nucleotides (nt) and non-enveloped icosahedral virions, 14 to 17 nm in diameter. Members of this family are constituted by 60 capsid protein subunits organised in a dodecahedral pentamer clustered unit [1]. Numerous circoviruses have been found in avian species, fish, insects, and mammals. Three species of circoviruses infect pigs: Porcine circovirus 1 (PCV1) was identified in a cell culture and it has not been associated with disease, PCV2 was identified in pigs with postweaning multisystemic wasting syndrome (PMWS) [2]. PCV2 was subsequently associated with other clinical diseases, now termed PCV2-related disease (PCVD) or porcine circovirus-associated disease (PCVAD), which includes PMWS, pneumonia, porcine dermatitis and nephropathy syndrome (PDNS), and reproductive failure. Disease induction requires infection with other microorganisms in addition to PCV2, for example, porcine parvovirus (PPV), porcine reproductive and respiratory syndrome virus (PRRSV), and *Mycoplasma hyopneumoniae* [3, 4]. PCV3 is a new member of the genus *Circovirus*, in the family *Circoviridae*, detected in a farm in North Carolina in 2017, with significant differences in the sequence when compared with PCV1 and PCV2, but more related to a bat-faeces associated circovirus [5, 6]. PCV-1 has a genome size ranging from 1758 to 1760 nt, while the circular genomes of PCV-2 and PCV-3 consist of 1766–1769 and 1999–2001 nt, respectively [1]. Sequence analysis showed that PCV3 is closely related to bat [7], and canine circoviruses [6]. Like all circoviruses, PCV3 contain three major open reading frames (ORF). ORF1 encodes the replicase protein of 297 amino acid (aa), ORF2 the capsid protein of 241 aa. ORF2 is in opposite orientation to ORF1. Since the start codon of ORF3 remains unclear, one initiation codon would result in a 231 amino acid (aa) protein, another in 177 aa protein [1]. For the protein encoded by ORF3 of PCV1 and PCV2 an apoptopic activity has been described, while its putative function in PCV3 is still unknown.

Meanwhile PCV3 has been detected in animals in the USA [5, 6], China [7–13], South Korea [14], Poland [15], Brazil [16], Thailand [17], Spain [18], Denmark [18], Sweden [19], Italy [18, 20], Russia [21] and Germany [22]. PCV3 was found to be associated with porcine dermatitis and nephropathy syndrome (PDNS), reproductive failure, and multisystemic inflammation [5, 6, 9], reproductive problems [5–7, 16, 20], cardiac and multisystemic inflammation [5], respiratory diseases [8, 9, 23] and congenital tremors in neonatal pigs [10]. In other cases PCV3 was found in animals without clinical signs of infection [24]. Since the pigs in most cases were co-infected with other porcine viruses, e.g., Torque teno sus virus 1 and 2 (TTsuV 1, TTsuV 2) [25], the pathogenicity of PCV3 warrants further investigations. In Swedish pigs, 70% of the PCV3-positive animals were positive for TTSuV1 and TTSuV2, 50% for porcine bocavirus (PBoV) [19].

When we started our study, it was unknown whether PCV3 can infect wild boars. We were analysing wild boars in two different urban forests in Berlin as well as in forests in Brandenburg, outside Berlin for the copy number of porcine endogenous retroviruses (PERVs) using droplet digital PCR (ddPCR). The samples had been taken between 2011 and 2015. The animals had been analysed genetically based on the analysis of 13 microsatellite loci and two major Bayesian Analysis of Population Structure (BAPS) clusters had been identified, the Grunewald BAPS cluster 1 and the Brandenburg BAPS cluster 2 (including also wild boars from Pankow) [26]. These forests were located in different parts of Berlin and Germany divided by the Berlin wall during the existence of two German states. This means the wild boars in East and West Berlin had no contact in the years from 1961 until 1989. Based on these genetic differences and the geographic isolation for a limited time, we expected interesting results concerning the copy number of PERV (to be published elsewhere). At the same time first results showing a high prevalence of PCV3 in domestic pigs (between 7 and 56% [1]) had been published, but it was still unknown whether PCV3 infects wild boars. So we started to screen these wild boars for PCV3. In the meantime PCV3 was found in Italian [27] as well as Spanish [28] wild boars. Our data demonstrate for the first time PCV3 in German wild boars and indicate that PCV3 is prevalent among Europe’s wild boars. The fact that PCV3 was present in genetically different populations which had been geographically isolated, suggests that the virus was introduced a long time before.

Worldwide, the population density of the wild boar seems to be increasing, which means a larger number of hosts available for infection with viruses as well as a higher contact rate between hosts [29]. Knowledge of infections and diseases in wildlife populations are not only important for livestock production (as a reservoir for domestic pigs) but also for public health.

## Materials and methods

### Animals

Wild boars were hunted in the city of Berlin as well as in adjoining parts of the federal State of Brandenburg, Germany. Between 1949 and 1989, two German states, the Federal Republic of Germany (FRG) and the German Democratic Republic (GDR) existed and Berlin was divided, whose Western part was surrounded by the 167-km-long Berlin wall. In total four genetic clusters of wild boars have been identified based on the analysis of 13 microsatellite loci, two within urban forests that belonged to former West-Berlin (Grunewald and Tegel) and two in the former Eastern part (Köpenick and Brandenburg including Pankow) [26]. We included samples of one Eastern and one Western cluster in this study: the Grunewald Bayesian Analysis of Population Structure (BAPS) cluster 1 and the Brandenburg BAPS cluster 2 (including also wild boars from Pankow) [26]. Spleens were sampled between 2011 and 2015 and stored frozen at − 20 °C.

### DNA isolation

DNA was extracted using the First-DNA all-tissue Kit (Gen-Ial GmbH, Troisdorf, Germany), following the manufacturer’s instructions.

### Real-time PCR, PCR

Quantitative real-time PCR [6] was used to detect PCV3 genomes in the isolated DNA of the wild boars. The real-time PCR was performed using specific primers and a probe (Table 1). The real-time PCR mixture contained 10 μl SensiFAST Probe No-Rox Mix (Bioline), 5 pmol from each primer, 5 pmol probe, 2 μl template DNA (150–300 ng) and sterile and distilled water to bring the final volume to 20 μl per sample. For amplification the Stratagene Mx3000P thermal cycler instrument (Agilent Technologies) was used with the following conditions: denaturation at 95 °C for 5 min and 45 cycles of amplification with denaturation at 94 °C for 15 s, annealing at 60 °C for 1 min and extension at 72 °C for 10 s. PCV1 and PCV2 were detected by PCR using specific primers (Table 1), amplified in a mastercycler pro S thermal cycler (Eppendorf) with the following condition: denaturation at 95 °C for 12 min and 42 cycles of amplification with denaturation at 95 °C for 20 s, annealing at 57 °C for 20 s and extension at 72 °C for 45 s followed by a final extension of 10 min at 72 °C The PCR mixture contained 1,25 U AmpliTaq Gold, 200 μM dNTPs (10 mM each), 10 pmol of each primer, 2 μL 10x PCR Gold buffer without MgCl_2_, 2 mM MgCl_2_, 1.5 μL template DNA (150 ng) and sterile and distilled water to bring the final volume to 20 μl per sample.

**Table 1:** Primers and probes used for PCV screening and sequencing

Primer, probe	Sequence	Accession number	Position (nt - nt)	Length of the amplicon (bp)	Reference
PCV3_real_FW	AGTGCTCCCCATTGAACG	KT869077	1427–1444	135	Palinski et al., 2016 [6]
PCV3_real_RV	ACACAGCCGTTACTTCAC		1561–1544		
PCV3_real_probe	FAM-ACCCCATGGCTCAACACATATGACC-BHQ1		1473–1449		
PCV3_seq2_FW	GTCGTCTTGGAGCCAAGTG		1609–1627	825	Palinski et al., 2016 [6]
PCV3_seq2_RV	CGACCAAATCCGGGTAAGC		433–415		
PCV1 fw (F41)	ATACGGTAGTATTGGAAAGGTAGGG		441–465	688	Mankertz et al., 2000 [42]
PCV1 rev (B42)	ACACTCGATAAGTATGTGGCCTTCT		1129–1106		
PCV2 fw (F66)	GGTTTGTAGCCTCAGCCAAAGC	KT868491.1	567–546	416	
PCV2 rev (B67)	GCACCTTCGGATATACTGTCAAGG		152–175		

### Cloning and sequencing

Amplicons were produced using a high fidelity PfuUltra II Fusion HS DNA polymerase (Agilent) and the forward primers PCV3_real_FW (1004 bp) or PCV3_seq2_FW (825 bp) and the reverse primer PCV3_seq2_RV (Table 1). The amplicons were cloned using the Zero Blunt™ TOPO™ PCR Cloning Kit and the pCR 4-Blunt TOPO™ vector (Thermo Fisher Scientific) and sequenced thereafter. In parallel, some amplicons were sequenced directly by Sanger sequencing.

### Ethics statement

All tissue samples were collected from wild boars hunted independent of the project; therefore no wild boars were harmed or killed for the project.

## Results

### Detection of PCV3 in Berlin wild boars

**Fig. 1:**
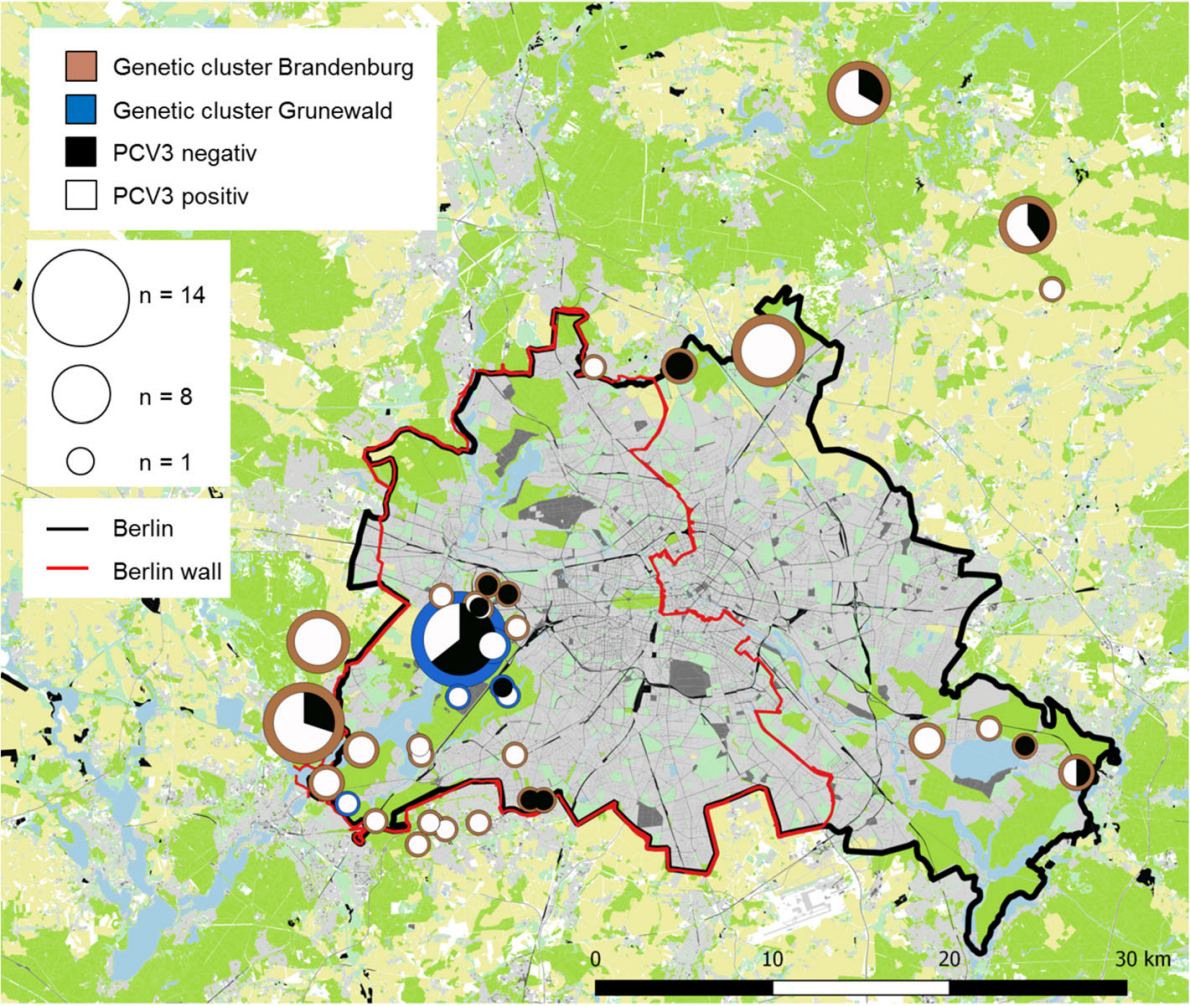
Prevalence of PCV3 and distribution of different genetic clusters of wild boars in and near Berlin. The size of the circles indicates the total number of investigated pigs, the percentage of PCV3-positive animals is shown in white inside the circle, the percentage of PCV3-negative animals in black. Two genetic clusters were identified, the Grunewald cluster (blue) and the Brandenburg cluster (brown). The border of Berlin (black line) is shown as well as the Berlin wall (red line) around the Western part of Berlin, which existed between 1961 and 1989. Around Berlin is the state Brandenburg. Background map: Habitat map of Berlin (modified after Stillfried et al. [43])

Based on the analysis of 13 microsatellite loci, samples could be grouped into two genetic clusters: Animals living in the Grunewald forest, in the former Western part of Berlin, belonged to the BAPS cluster 1, while animals in the other regions and mainly in Brandenburg belonged to the BAPS cluster 2 [26] (Fig. 1). When animals from both clusters were tested for PCV3 using a real-time PCR, 10 of 20 animals (50%) in the Grunewald cluster and 16 of 69 animals (23%) in the Brandenburg cluster were found positive (Fig. 1, Table 2).

**Table 2:** Results of the PCV testing

Region	Previously to	Genetical data	PCV3 infection (number positive/ number total) (%)	PCV1 Infection (number positive/number total) (%)	PCV2 Infection (number positive/number total) (%)	PCV1 and PCV3 double infection (number positive/number total) (%)	PCV2 and PCV3 double infection (number positive/number total) (%)	PCV1, PCV2, PCV3 triple infection (%)
Grunewald	West-Berlin	13 microsatellite loci, BAPS cluster 1	10/20 (50%)	2/16^a^ (12.5%)	2/16 (12.5%)	2/16 (12.5%)	2/16 (12.5%)	1/16 (6.25%)
Brandenburg	East-Germany	13 microsatellite loci, BAPS cluster 2	16/69 (23%)	7/40^b^ (17.5%)	4/40 (10%)	7/40 (17.5%)	3/40 (7.5%)	1/40 (2.5%)

^a^only 16 animals of 20 have been tested; ^b^only 40 of 69 animals have been tested

Viruses from both clusters (six viruses from animals of the Grunewald BAPS cluster and four viruses from animals of the Brandenburg BAPS cluster) were amplified, cloned and sequenced (Table 3). Nine of the ten viruses belonged to the subtype PCV3b according to the classification by Fux et al. [22] based on the specific amino acids in position 24, 27 and 77 of the ORF2 protein (Table 3), one virus, from wild boar 20 of the Grunewald cluster, belonged to the subtype PCV3a having amino acid lysine (K) instead of arginine (R) in position 27 of the ORF2 protein.

**Table 3:** Classification of the sequenced PCV3 from wild boars (WB) in and around Berlin based on the definition by Fux et al. [22]

subtype	genetic cluster	aa24	aa27	aa77	aa150
a1	n/a	V	K	S	I
a2	n/a	A/V	K	S	I
b1	n/a	A	R	S	I
b2	n/a	A	R	T	L
WB 12	GW	A	R	S	n.t.
WB 13	GW	A	R	S	n.t.
WB 14	GW	A	R	S	n.t.
WB 17	GW	A	R	S	n.t.
WB 20	GW	A	K	S	I
WB 111	BB	A	R	S	I
WB 212	GW	A	R	S	I
WB 323	BB	A	R	S	n.t.
WB 347	BE	A	R	S	I
WB 349	BE	A	R	S	I

*n/a* not applicable, *aa* amino acids, *V* valine, *K* lysine, *I* isoleucine, *L* leucine, *R*. arginine, *WB* wild boar, *GW* Grunewald cluster, *BB* Brandenburg cluster, *n.t*., not tested

Sequencing repeatedly we found only PCV3b or in a single case only PCV3a in the wild boars.

### Detection of PCV1 and PCV2 in Berlin wild boars

In order to investigate whether the wild boars in and around Berlin were also infected with PCV1 and PCV2, PCR analyses were performed using specific primers (Table 1). 12.5% of the analysed 16 animals in the Grunewald BAPS cluster and 17.5% of the tested 40 animals of the Brandenburg BAPS cluster were positive for PCV1 (Table 2). 12.5 and 10% were positive for PCV2 in the Grunewald and Brandenburg cluster, respectively. The percentage of the PCV1 and PCV3 double infected animals were 12.5 and 17.5%, that of PCV2 and PCV3 double infected animals 12.5 and 7.5% and the percentage of triple infected was 6.25 and 2.5% (Table 2).

## Discussion

Here, the first description of PCV3 in German wild boars is given. Up to 50% of the animals in the Grunewald cluster are positive, whereas the percentage in the Brandenburg cluster was lower (23%). PCV3 has been described recently in Italian wild boars with a similar incidence (33%) [27], and after submission of our manuscript, 42.66% PCV3-positive wild boars had been described in Northeastern Spain [28]. Our results confirm that PCV3 is not only present in domestic pigs, but also in wild boars and that PCV3 is prevalent among Europe’s wild boars for many years. PCV3 is distributed worldwide in domestic pigs, with very similar viruses in different places, possibly due to the transportation of the animals from one country to another. At present it is unclear whether the virus was introduced into the production pigs and wild boars at nearly the same time, or whether production pigs distributed the virus worldwide and also infected wild boars or whether wild boars were infected first, transmitting the virus to the production pigs. The closest ancestor of PCV3 was found to be within the clade 1 of bat circoviruses which were isolated in China between 2011 and 2013 [30]. More detailed investigations allowed describing subclades PCV3a and PCV3b and division of PCV3a into PCV3a-1 and PCV3a-2 [28]. Amino acid sites S122A, A320V (amino acid site 24 of the ORF2 protein) and 323 were crucial to discriminate PCV3a and PCV3b. The viruses from the Berlin wild boars we sequenced belong predominantly to the PCV3b clade, but in one animal also PCV3a was found (Table 3). It remains unknown whether in some animals a co-infection of PCV3a and PCV3b took place. Our data correlated with the data by Fux et al. [22] showing a higher number of PCV3b compared with PCV3a in farm animals in Germany. Since the nucleotide identity among these sequences is really high (greater 97%) [22, 31], it is not important to determine genotypes or groupings.

The prevalence of PCV3a and PCV3b in all countries is remarkable and the worldwide circulation of PCV3 leading to multiple introductions events followed by local evolution appears the most likely scenario to explain this [32]. Furthermore, the demonstration of a PCV3 infection in asymptomatic animals, could have favoured the undetected and uncontrolled circulation of the virus [24]. A similar scenario had also been proposed for the distribution of PCV2 [29].

There are data showing that PCV3 did not emerge through recombination events among currently known circoviruses and that its speciation is not a recent evolutionary event [32]. The most common recent ancestor analysis suggests that PCV3 lineages have emerged over the past 50 years. PCV3 is not genetically closely related with other porcine circoviruses and it has been evolving undetected for some time in the swine population. The finding of groups of genetically related isolates of PCV3 originated from different countries that may be associated with dispersal routes, suggesting that PCV3 has already been circulating in pig-producing countries for some time before its first detection [32]. Analysing the trade flows of live pigs in the years 2006–2016 showed mainly export flows from the USA to Brazil, China, and Korea. But these countries also exported pigs to other countries, leading to an intense movement of live pigs among producing countries [32].

Although it is still unclear whether PCV3 is associated with a disease in pigs, the absence of disease in some infected pigs [24] suggests that similarly to PCV2 additional factors or co-infections with other microorganisms are required to induce severe diseases. PCV2 is necessary but not sufficient for the induction of PCVD. PCVD is a whole complex of diseases including PCV2-systemic disease (PCV2-SD, previously called PMWS), PCV2-subclinical infection (PCV2-SI), PCV2-reproductive disease (PCV2-RD), and PDNS. Some purported risk factors include co-infection with other viruses, e.g., PRRSV, which causes the porcine reproductive and respiratory syndrome associated with reproductive failure in breeding stocks and respiratory tract illness in young pigs. Co-infection with porcine parvovirus may also contribute to PCVD. As mentioned in the introduction, PCV3 was found to be associated with PDNS, reproductive failure, and multisystemic inflammation [5, 6, 9], reproductive problems [5–7, 16, 20], cardiac and multisystemic inflammation [5], respiratory diseases [8, 9, 23] and congenital tremors in neonatal pigs [10]. However, in some cases, PCV3 was found in animals without clinical signs of infection [24]. Animals generated for xenotransplantation purposes should be not only healthy but also free from PCV3 and other circoviruses [33]. Like PCV2, PCV3 may be an immunosuppressive virus and it is still unclear whether subclinical infections of pigs may decrease the functionality of the organs required for transplantation. However, to eliminate the virus from the animals will be difficult, since PCV3 is transmitted by the colostrum [34].

Numerous viruses have been found in wild boars, including suid alphaherpesvirus 1 (pseudorabies virus), a pestivirus causing classical swine fever, PRRSV, PCV2 and many others [35]. PCV2 has been previously described in German wild boars [36]. PCV2 seroprevalence has been reported to be around 30–40% in Belgian and Spanish wild boars [37]. Using PCR, 20% of Hungarian wild boars have been found positive for PCV2 [38]. Some of the animals were suffering from PMWS [38]. It seems likely that the origin of PCV2 infection in wild boars could be through contact with domestic pigs, not least because of the high PCV2 infection rate in pig herds. PCV2 has also been found in slaughterhouse pigs in Berlin and in Göttingen Minipigs [39]. Göttingen Minipigs had been recently used in preclinical pig islet cell transplantations into non-human primates [40].

In this context, it is of interest that some of the investigated German wild boars were infected in addition to PCV3 with PCV1 or PCV2 or even with all three viruses. However we do not have data on the health status of the wild boars and cannot judge what happens in the case of double or triple infections. Double infection with PCV2 and PCV3 has also been found in diseased domestic pigs in the Henan province in China (around 30% of the animals) [41], in another study 22.3% of 76 clinical PCV3-positive samples were found to be co-infected with PCV2 [44].

## Conclusion

PCV3 is widely distributed in wild boars in and around Berlin, which form two genetically distinct and geographically coherent clusters and which were divided during the existence of the Berlin wall. In both clusters PCV3b was detected, and in one cluster also PCV3a. For the first time, also double and triple infections in wild boars with PCV1 and PCV2 were observed. The methods used will be needed to screen for circoviruses in pigs genetically modified for application in xenotransplantation.
